# Coarse-Grained Hawkes Processes

**DOI:** 10.3390/e27060555

**Published:** 2025-05-25

**Authors:** Shinsuke Koyama

**Affiliations:** Department of Interdisciplinary Statistical Mathematics, The Institute of Statistical Mathematics, Tokyo 190-8562, Japan; skoyama@ism.ac.jp

**Keywords:** Hawkes process, aggregated data, count time series, coarse-grained modeling

## Abstract

When analyzing real-world event data, it is often the case that bin-count processes are observed instead of precise event time-stamps along a continuous timeline, owing to practical limitations in measurement accuracy. In this work, we propose a modeling framework for aggregated event data generated by multivariate Hawkes processes. The introduced model, termed the coarse-grained Hawkes process, effectively captures the second-order statistical characteristics of the bin-count representation of the Hawkes process, particularly when the bin size is large relative to the typical support of the excitation kernel. Building upon this model, we develop a method for inferring the underlying Hawkes process from bin-count observations, and demonstrate through simulation studies that the proposed approach performs comparably to, or even surpasses, existing techniques, while maintaining computational efficiency in parameter estimation.

## 1. Introduction

Generally, past events in natural and social systems facilitate the occurrence of future events through a self-exciting mechanism. The Hawkes process [1,2], a class of self-exciting point processes, has become a popular tool for modeling such processes continuously. Its defining intensity function, or instantaneous event rate, is composed of a baseline rate augmented by the cumulative influence of prior events, thereby capturing the self-exciting mechanism wherein each event elevates the probability of subsequent occurrences. Owing to the prevalence of self-excitation across diverse domains, the Hawkes process has been extensively employed in a wide range of disciplines, including seismology [3,4], neurophysiology [5,6], genomics [7], finance [8,9], social media analytics [10,11,12], criminology [13,14], terrorism studies [15], and traffic incident analysis [16].

The Hawkes process is applicable to scenarios wherein individual events are distinguishable, as it characterizes a series of discrete occurrences along a continuous timeline. Nevertheless, it proves insufficient for modeling count-based sequences in which the precise timing of events is unobserved and only their aggregated counts within successive intervals are available. A representative case arises in epidemiological studies, where individual infection events are not monitored in real time, but rather, the daily incidence rates are recorded. Consequently, there is a need to develop a count-based time series model that retains the salient features of the Hawkes process for such contexts.

Multiple methodologies have been proposed for fitting the Hawkes process to bin-count data. Kirchner demonstrated that the distribution of bin-count sequences generated by Hawkes processes can be effectively approximated using an integer-valued autoregressive (INAR) model, wherein conditional least squares estimation is employed to infer the underlying Hawkes process [17,18]. Shlomovich et al. introduced a Monte Carlo expectation-maximization (MC-EM) framework, which integrates an efficient sampling algorithm for the latent event times with an EM procedure to maximize the likelihood function [19,20]. Similarly, Chen et al. developed a Pseudo-Marginal Metropolis–Hastings (PMMH) algorithm for maximum likelihood estimation, wherein the likelihood is approximated via a sequential Monte Carlo approach [21]. Alternatively, Cheysson and Lang advocated for a spectral estimation technique grounded in the Whittle likelihood within the univariate context [22].

Although Kirchner’s approach provides consistent and asymptotically normal estimators for the underlying Hawkes process as the bin width approaches zero, it introduces bias when the bin size exceeds the typical support of the excitation function, due to the INAR model’s neglect of intra-bin excitation dynamics. In contrast, the MC-EM, PMMH, and spectral estimation methods yield less biased results by accounting for excitation effects occurring within each bin. Nonetheless, the MC-EM and PMMH techniques are computationally intensive, whereas the spectral method is validated only in the univariate setting.

Accordingly, the objective of this study is to develop a computationally efficient methodology that extends naturally to the multivariate setting. Our proposed approach involves constructing a count-based time series model that approximates the bin-count sequence generated by Hawkes processes, upon which an estimation procedure is built. Unlike Kirchner’s method, which relies on a straightforward discretization, namely, evaluating the intensity function at discrete time points, to relate the Hawkes and INAR processes [17], our model is derived through a coarse graining procedure. The resulting framework, termed the coarse-grained Hawkes process, is defined in a conceptually simple and analytically tractable form, while effectively preserving the second-order statistical characteristics of the bin-count Hawkes process, even in regimes where the bin size is large relative to the excitation function’s effective range.

The structure of this paper is as follows. Section 2 provides a concise overview of Hawkes processes. Section 3 introduces the coarse-grained Hawkes process along with the proposed estimation methodology. In Section 4, we assess the approximation accuracy of the coarse-grained Hawkes process in representing the bin-count Hawkes process. Additionally, a simulation study is conducted to benchmark the performance of the proposed estimation technique against existing approaches. Section 5 concludes with a discussion of the findings.

## 2. Review of Hawkes Processes

In this section, we present a brief review of Hawkes processes. Let ${\left\{t_{i}\right\}}_{i\in N}$ denote a sequence of nonnegative random variables representing the occurrence times of events on $R_{+}$, satisfying $t_{i}<t_{i+1}$ for i∈N. Define the associated counting process by $N\left(t\right)=\sum_{i\in N}1_{t_{i}\leq t}$, and let $H_{t}=\left\{N\left(u\right):u<t\right\}$ denote the history of events up to, but not including, time *t*. We consider a point process such that

$$\begin{array}{l} P(N\left(t+\delta t\right)-N\left(t\right)=1|H_{t})=\lambda (t)\delta t+o(\delta t),\\ P(N\left(t+\delta t\right)-N\left(t\right)>1|H_{t})=o(\delta t), \end{array}$$

as δt→0, where λ(t) is the conditional intensity function that uniquely characterizes the point process. A univariate Hawkes process is then defined by the conditional intensity function

$$\lambda \left(t\right)=\mu +\int_{0}^{t}\phi \left(t-u\right)dN\left(u\right),$$

where μ≥0 is the baseline intensity and ϕ(·) is a nonnegative excitation kernel satisfying ϕ(t)=0 for t<0 [1]. This formulation captures the self-exciting nature of the process, wherein the intensity at any given time depends on the historical sequence of events. The branching ratio is defined as

$$\alpha :=\int_{0}^{\infty }\phi \left(t\right)dt,$$

which quantifies the expected number of subsequent events triggered by a single occurrence. The process admits a stationary distribution provided that α<1.

The univariate Hawkes process naturally generalizes to the multivariate case [1,2]. For d∈N, a *d*-dimensional Hawkes process $N\left(t\right)={\left(N_{1}\left(t\right),\ldots ,N_{d}\left(t\right)\right)}^{T}$ comprises *d* jointly defined point processes on $R_{+}$. The corresponding vector-valued conditional intensity function is given by

$$\lambda \left(t\right)=\mu +\int_{0}^{t}\Phi \left(t-u\right)dN\left(u\right),$$

where $\mu ={\left(\mu_{1},\ldots ,\mu_{d}\right)}^{T}\in R_{\geq 0}^{d}$ is the baseline intensity vector, and the excitation kernel $\Phi ={\left(\phi_{ij}\right)}_{1\leq i,j\leq d}$ is a matrix-valued function with nonnegative entries satisfying $\phi_{ij}\left(t\right)=0$ for t<0. Each entry $\phi_{ij}\left(\cdot \right)$ describes the influence of process *j* on the intensity of process *i*, thus capturing both self-excitation and mutual excitation among the components. The multivariate Hawkes process is asymptotically stationary if the spectral radius of the branching matrix

$$A:=\int_{0}^{\infty }\Phi \left(t\right)dt$$

is strictly less than one.

Finally, we briefly recall the martingale properties of point processes that are instrumental for our methodological developments. For a comprehensive treatment, see [23]. Define the process M(t)=N(t)−Λ(t), where $\Lambda \left(t\right)=\int_{0}^{t}\lambda \left(u\right)du$. Then, M(t) is a martingale with respect to the filtration $H_{t}$, satisfying the property that the conditional expectation of its increment is zero:

$$E\left[M\left(t\right)-M\left(s\right)|H_{s}\right]=0,\;t>s.$$

Moreover, the conditional covariance matrix of the martingale increment is given by

$$E\left[\left(M\left(t\right)-M\left(s\right)\right){\left(M\left(t\right)-M\left(s\right)\right)}^{T}|H_{s}\right]=\mathrm{diag}\left(E[\Lambda \left(t\right)-\Lambda \left(s\right)|H_{s}]\right),\;t>s,$$

which follows from the quadratic variation of the martingale.

## 3. Coarse-Grained Hawkes Process

### 3.1. Motivation

We consider a scenario wherein the exact timing of individual events is unobserved; instead, we observe an aggregated count of these latent continuous-time events within discrete time intervals. We define

$$X_{n}=N\left(n\Delta t\right)-N\left(\left(n-1\right)\Delta t\right),\;n\in N,$$

as the bin-wise event counts over intervals of size Δt. When $\left\{N\left(t\right):t\in R_{+}\right\}$ constitutes a Hawkes process, the corresponding discrete-time vector process $\left\{X_{n}:n\in N\right\}$ is referred to as the binned Hawkes process [19,20].

Although a closed-form representation of the probability distribution of binned Hawkes processes is not available, an approximate formulation can be derived by discretizing the conditional intensity function as follows:

(1) $$\lambda_{n}={\left(\lambda_{1n},\ldots ,\lambda_{dn}\right)}^{T}=\mu \Delta t+\sum_{k=1}^{n-1}\Phi \left(k\Delta t\right)\Delta tX_{k},$$

and assuming that the bin counts follow Poisson distributions:

(2) $$X_{n}|X_{1},\ldots ,X_{n-1}∼\prod_{j=1}^{d}\mathrm{Poisson}\left(\lambda_{jn}\right).$$

This method, known as the binned Poisson approximation, converges in distribution to the true Hawkes process as Δ→0. However, its accuracy degrades with larger Δt due to discretization errors in (1) and the conditional independence assumption in (2), which neglects the intra-bin excitation effect. Nonetheless, it facilitates a tractable estimation procedure: the log-likelihood of the sequence $X_{1},\ldots ,X_{n}$ is given by

$$\log P\left(X_{1},\ldots ,X_{n}\right)=\sum_{k=1}^{n}\sum_{j=1}^{d}\left(X_{jk}\log\lambda_{jk}-\lambda_{jk}\right).$$

Parameter estimation is then performed via maximization of this log-likelihood function [19,20,24].

In light of the limitations of this approximation for larger Δt, we propose an alternative count time series model that more accurately approximates the binned Hawkes process while retaining computational simplicity for inference. The underlying heuristic is elucidated in the following discussion.

The central idea is to replace the crude discretization with the expected value of the conditional intensity integrated over each time bin. The expected count within the *n*th bin of process *i* is $\int_{(n-1)\Delta t}^{n\Delta t}\lambda_{i}\left(t\right)dt$, which depends on the trajectory {N(u):0<u≤nΔt}. Consequently, we consider the conditional expectation, given the bin counts $\left\{X_{1},\ldots ,X_{n}\right\}$:

(3) $$\begin{array}{cc} \lambda_{in}\; & =E\left[\int_{(n-1)\Delta t}^{n\Delta t}\lambda_{i}\left(t\right)dt|X_{1},\ldots ,X_{n}\right]\\ \; & =\mu_{i}\Delta t+\sum_{k=1}^{n-1}\sum_{j=1}^{d}E\left[\int_{(n-1)\Delta t}^{n\Delta t}\left(\int_{(k-1)\Delta t}^{k\Delta t}\phi_{ij}\left(t-u\right)dN_{j}\left(u\right)\right)dt|X_{jk}\right]\\ \; & +\sum_{j=1}^{d}E\left[\int_{(n-1)\Delta t}^{n\Delta t}\left(\int_{(n-1)\Delta t}^{t}\phi_{ij}\left(t-u\right)dN_{j}\left(u\right)\right)dt|X_{jn}\right]. \end{array}$$

As these conditional expectations do not admit closed-form expressions, we approximate them under the assumption that event times are uniformly distributed within each bin. Accordingly, the second term on the right-hand side of (3) is approximated as

$$\begin{array}{cc} \; & E\left[\int_{(n-1)\Delta t}^{n\Delta t}\left(\int_{(k-1)\Delta t}^{k\Delta t}\phi_{ij}\left(t-u\right)dN_{j}\left(u\right)\right)dt|X_{jk}\right]\\ \; & \approx \int_{(k-1)\Delta t}^{k\Delta t}\cdots \int_{(k-1)\Delta t}^{k\Delta t}\frac{1}{\Delta t^{X_{jk}}}\left(\sum_{i=1}^{X_{jk}}\int_{(n-1)\Delta t}^{n\Delta t}\phi_{ij}\left(t-t_{i}\right)dt\right)dt_{1}\ldots dt_{X_{jk}}\\ \; & =\phi_{n-k}^{ij}X_{jk}, \end{array}$$

where $\phi_{n-k}^{ij}$ denotes the coarse-grained kernel, defined as

(4) $$\phi_{n-k}^{ij}=\frac{1}{\Delta t}\int_{(k-1)\Delta t}^{k\Delta t}\int_{(n-1)\Delta t}^{n\Delta t}\phi_{ij}\left(t-u\right)dudt.$$

Similarly, the third term on the right-hand side of (3) is approximated by

$$\begin{array}{cc} \; & E\left[\int_{(n-1)\Delta t}^{n\Delta t}\left(\int_{(n-1)\Delta t}^{t}\phi_{ij}\left(t-u\right)dN_{j}\left(u\right)\right)dt|X_{jn}\right]\\ \; & =E\left[\int_{(n-1)\Delta t}^{n\Delta t}\left(\int_{(n-1)\Delta t}^{n\Delta t}\phi_{ij}\left(t-u\right)dN_{j}\left(u\right)\right)dt|X_{jn}\right]\approx \phi_{0}^{ij}X_{jn}, \end{array}$$

where we have utilized the causal property $\phi_{ij}\left(t\right)=0$ for t<0. Substituting these approximations back into (3) yields

(5) $$\lambda_{in}\approx \mu_{i}\Delta t+\sum_{k=1}^{n}\sum_{j=1}^{d}\phi_{n-k}^{ij}X_{jk}.$$

In contrast to the formulation in (1), Equation (5) incorporates the coarse-grained kernel, capturing both inter-bin excitation and intra-bin self-excitation effects through the terms $\phi_{0}^{ij}X_{jn}$ (1≤i,j≤d). Building upon this approximation, we will formally define the coarse-grained Hawkes process in the subsequent section.

### 3.2. Definition

Firstly, we formally define the coarse-grained kernel, previously derived heuristically in (4).

**Definition** **1.** *Let ϕ(t) be a nonnegative excitation kernel defined on the real line, satisfying ϕ(t)=0 for t<0. The coarse-grained kernel ${\left\{\phi_{k}\right\}}_{k\in N_{0}}$ with bin size Δt>0 is defined by*

(6) $$\phi_{k}=\left\{\begin{array}{cc} \xi_{0}, & k=0,\\ \xi_{k}-\xi_{k-1}, & k=1,2,\ldots , \end{array}\right.$$

*where*

(7) $$\xi_{k}=\frac{1}{\Delta t}\int_{k\Delta t}^{(k+1)\Delta t}\left(\int_{0}^{t}\phi \left(u\right)du\right)dt.$$

It can be readily verified that the formulation (6) and (7) coincides with the expression in (4).

**Lemma** **1.** *The coarse-grained kernel satisfies*

$$\begin{array}{c} \sum_{k=0}^{\infty }\phi_{k}=\int_{0}^{\infty }\phi \left(u\right)du=:\alpha . \end{array}$$

*Moreover, it holds that $\phi_{0}<\alpha$.*

**Proof.**  See Appendix A.1.    □

Lemma 1 guarantees that the total mass of the coarse-grained kernel equals the integral of the excitation kernel, i.e., the branching ratio. Furthermore, it ensures that the kernel does not collapse to the degenerate case $\phi_{0}=\alpha$ as long as Δt<∞.

Based on the coarse-grained kernel, we introduce the coarse-grained Hawkes process. Consider a probability space equipped with a sequence of *d*-dimensional, integer-valued random vectors ${\left\{{\tilde{X}}_{k}\right\}}_{k\in Z}$. Define the history up to bin *k* as $H_{k}=\left\{{\tilde{X}}_{j}:j\leq k\right\}$. We consider the *d*-dimensional coarse-grained process given in (5):

(8) $$\lambda_{n}=\mu \Delta t+\sum_{k\leq n}\Phi_{n-k}{\tilde{X}}_{k},\;n\in Z,$$

where $\mu \in R_{\geq 0}^{d}$ is the baseline intensity vector, and each $\Phi_{n-k}={\left(\phi_{n-k}^{ij}\right)}_{1\leq i,j\leq d}$ is a matrix whose elements are given by the coarse-grained kernels for bin size Δt. Define the residual process as

(9) $$\Delta M_{n}={\tilde{X}}_{n}-\lambda_{n},\;n\in Z,$$

and impose the following conditional moment properties, analogous to the martingale conditions in point process theory:

(10) $$E[\Delta M_{n}|H_{n-1}]=0,$$

(11) $$E\left[\Delta M_{n}\Delta M_{n}^{T}|H_{n-1}\right]=\mathrm{diag}\left(E\left[\lambda_{n}|H_{n-1}\right]\right).$$

Importantly, these conditional moment properties do not fully specify the probability law of the process, but constrains its behavior through second-order statistical structure. Consequently, multiple processes may exist that fulfill these conditions. The coarse-grained Hawkes process is thus defined as the equivalence class of sequences $\left\{{\tilde{X}}_{k}\right\}$ satisfying (8)–(11).

The term $\Phi_{0}{\tilde{X}}_{n}$ on the right-hand side of (8) encapsulates the effect of intra-bin excitation, which induces both cross-correlations and overdispersion in the count statistics. Assuming that the spectral radius of $\Phi_{0}$ is strictly less than one, the conditional expectation and covariance matrix of ${\tilde{X}}_{n}$ given $H_{n-1}$ are, respectively

(12) $$E\left[{\tilde{X}}_{n}|H_{n-1}\right]={\left(I-\Phi_{0}\right)}^{-1}\left(\mu \Delta t+\sum_{k\leq n-1}\Phi_{n-k}{\tilde{X}}_{k}\right)=:\lambda_{n}^{*},$$

(13) $$\mathrm{Var}\left({\tilde{X}}_{n}|H_{n-1}\right)={\left(I-\Phi_{0}\right)}^{-1}\mathrm{diag}\left(\lambda_{n}^{*}\right){\left(I-\Phi_{0}^{T}\right)}^{-1},$$

where *I* denotes the d×d identity matrix. Derivations of these expressions are provided in Appendix A.2. Due to the presence of the matrix factor ${\left(I-\Phi_{0}\right)}^{-1}$, the conditional covariance matrix is generally nondiagonal, capturing cross-correlations, and its diagonal elements exceed those of $\lambda_{n}^{*}$, reflecting overdispersion. When $\Phi_{0}$ is the zero matrix, implying the absence of intra-bin excitation, the conditional variance reduces to $\mathrm{Var}\left({\tilde{X}}_{n}|H_{n-1}\right)=\mathrm{diag}\left(\lambda_{n}^{*}\right)$, corresponding to the conditional independence of Poisson-distributed counts.

### 3.3. Stationary Process

We investigate the coarse-grained Hawkes process under the assumption of stationarity and derive its second-order statistical properties. Assuming stationarity, Equations (8) and (10) yield

(14) $$E\left[{\tilde{X}}_{n}\right]={\left(I-A\right)}^{-1}\mu \Delta t=:\lambda ,\;n\in Z,$$

where

$$A:=\sum_{n=0}^{\infty }\Phi_{n}$$

denotes the branching ratio matrix. Consequently, the spectral radius of *A* must be strictly less than one.

To derive the second-order moment of the stationary coarse-grained Hawkes process, we first establish a white noise property of the residual process.

**Lemma** **2.** *Let $\left\{{\tilde{X}}_{n}\right\}$ denote a coarse-grained Hawkes process whose branching ratio matrix has a spectral radius less than one. Then, the residual process (9) forms a stationary sequence satisfying $E[\Delta M_{n}]=0$, n∈Z, and*

(15) $$E\left[\Delta M_{n}\Delta M_{n^{\prime }}^{T}\right]=\delta_{nn^{\prime }}\mathrm{diag}\left(\lambda \right),\;n,n^{\prime }\in Z,$$

*where $\delta_{nn^{\prime }}$ denotes the Kronecker delta.*

**Proof.**  See Appendix A.3.    □

As a consequence of the white noise structure of the residual process, the stationary coarse-grained Hawkes process admits a moving average representation of infinite order,

(16) $${\tilde{X}}_{n}-\lambda =\sum_{k=0}^{\infty }\Psi_{k}\Delta M_{n-k},$$

where the effective kernel matrices $\Psi_{k}$ are defined by

$$\Psi_{k}=\sum_{j=0}^{\infty }{\left(\Phi^{\left(*j\right)}\right)}_{k},$$

with $\Phi^{\left(*j\right)}$ denoting the *j*-fold convolution of Φ, and ${\left(\Phi^{\left(*0\right)}\right)}_{k}=\delta_{k0}I$. It is worth noting that the branching ratio matrix can be expressed in terms of the effective kernel matrices as follows:

$$A=I-{\left(\sum_{n=0}^{\infty }\Psi_{n}\right)}^{-1},$$

thereby establishing a relationship between the branching ratio and the cumulative influence of preceding events. The detailed derivation is presented in Appendix A.4. Using the representation (16), the autocovariance structure is obtained analogously to linear time series models.

**Theorem** **1.** *Let $\left\{{\tilde{X}}_{n}\right\}$ be a coarse-grained Hawkes process with a branching ratio matrix whose spectral radius is less than one. Then, the autocovariance of $\left\{{\tilde{X}}_{n}\right\}$ is given by*

$$R_{j}:=\mathrm{Cov}\left({\tilde{X}}_{n},{\tilde{X}}_{n+j}\right)=\sum_{l=0}^{\infty }\Psi_{l-j}\mathrm{diag}\left(\lambda \right)\Psi_{l}^{T},\;j\in Z.$$

**Proof.**  See Appendix A.5.    □

The spectral density matrix of the stationary coarse-grained Hawkes process is obtained by taking the Fourier transform of the autocovariance sequence:

$$F_{\Delta t}^{\left(cg\right)}\left(\omega \right)=\frac{1}{2\pi }\sum_{n=-\infty }^{\infty }R_{n}e^{-i\omega n}=\frac{1}{2\pi }\hat{\Psi }\left(-\omega \right)\mathrm{diag}\left(\lambda \right){\hat{\Psi }}^{T}\left(\omega \right),$$

where $\hat{\Psi }\left(\omega \right)=\sum_{n=0}^{\infty }\Psi_{n}e^{-i\omega n}$ is the Fourier transform of the effective kernel matrix. Alternatively, it can be expressed via the Fourier transform of the coarse-grained kernel matrix, $\hat{\Phi }\left(\omega \right)=\sum_{n=0}^{\infty }\Phi_{n}e^{-i\omega n}$, as

(17) $$\begin{array}{c} F_{\Delta t}^{\left(cg\right)}\left(\omega \right)=\frac{1}{2\pi }{\left(I-\hat{\Phi }\left(-\omega \right)\right)}^{-1}\mathrm{diag}\left(\lambda \right){\left(I-{\hat{\Phi }}^{T}\left(\omega \right)\right)}^{-1}, \end{array}$$

utilizing the identity $\hat{\Psi }\left(\omega \right)={\left(I-\hat{\Phi }\left(\omega \right)\right)}^{-1}$.

In summary, under the condition that the spectral radius of the branching ratio matrix is less than one, the expected value of the process remains constant over time, and its autocovariance depends solely on the lag between observations, not their absolute positions in time. Accordingly, the coarse-grained Hawkes process is weakly stationary.

### 3.4. Approximation to Hawkes Process

We now establish a rigorous connection between the original Hawkes process and its coarse-grained counterpart. Specifically, we examine the second-order statistical properties of both processes under the assumption of stationarity. A summary of the statistical properties of the stationary Hawkes process is provided in Appendix B.

Since the sum of the coarse-grained kernel coincides with the integral of the excitation kernel (see Lemma 1), the branching ratio matrices, and consequently, the conditions for stationarity, are identical for both processes. Under stationarity, the expected event counts of the coarse-grained Hawkes process (14) coincide with those of the original Hawkes process (A7).

We proceed to compare the spectral density matrices of the two processes, focusing in particular on the convergence behavior of the spectral density of the coarse-grained process toward that of the original Hawkes process.

**Theorem** **2.** *The spectral density matrix of the coarse-grained Hawkes process satisfies*

(18) $$F_{\Delta t}^{\left(cg\right)}\left(\nu \Delta t\right)=F^{\left(hw\right)}\left(\nu \right)\Delta t+O\left({\Delta t}^{3}\right),$$

*as Δt→0, where $F^{\left(hw\right)}\left(\nu \right)$ denotes the spectral density matrix (A6) of the original Hawkes process.*

**Proof.**  See Appendix A.6.    □

Considering the binned Hawkes process, its spectral density matrix $F_{\Delta t}^{\left(hw\right)}\left(\omega \right)$ behaves as (see Appendix B)

$$F_{\Delta t}^{\left(hw\right)}\left(\nu \Delta t\right)=F^{\left(hw\right)}\left(\nu \right)\Delta t+O\left(\Delta t^{3}\right),$$

as Δt→0, which matches the expansion in (18) up to second-order terms in Δt. This leads to the following corollary.

**Corollary** **1.** *The spectral density matrix of the coarse-grained Hawkes process approximates that of the binned Hawkes process to third-order accuracy as*

$$F_{\Delta t}^{\left(cg\right)}\left(\omega \right)=F_{\Delta t}^{\left(hw\right)}\left(\omega \right)+O\left(\Delta t^{3}\right),$$

*as Δt→0.*

For comparative purposes, consider the binned Poisson approximation defined by Equations (1) and (2). Its spectral density matrix $F_{\Delta t}^{\left(po\right)}\left(\omega \right)$ behaves as (see Appendix A.6)

$$F_{\Delta t}^{\left(po\right)}\left(\omega \right)=F_{\Delta t}^{\left(hw\right)}\left(\omega \right)+O\left(\Delta t^{2}\right).$$

Therefore, the coarse-grained Hawkes process yields a spectral approximation to the binned Hawkes process that is accurate to a higher order than the binned Poisson approximation.

### 3.5. Parameter Estimation Method

We address the problem of estimating the parameters of a Hawkes process from binned count data. Assume that we observe a sequence of binned event counts $\left\{X_{1},\ldots ,X_{n}\right\}$ of length *n* generated by a *d*-dimensional Hawkes process, whose excitation kernel matrices $\Phi_{k}\left(\theta \right)$ are specified by a parametric form with an unknown parameter vector θ. Since the likelihood function of the coarse-grained Hawkes process is unavailable due to the absence of a fully specified probability law, we propose a parameter estimation method for θ grounded in the second-order statistical properties of the coarse-grained Hawkes process.

To this end, we utilize the AR(*∞*) representation of the coarse-grained Hawkes process (see Appendix A.4),

$${\tilde{X}}_{n}-\lambda =\sum_{k=1}^{\infty }\Phi_{k}^{*}\left(\theta \right)\left({\tilde{X}}_{n-k}-\lambda \right)+\Delta M_{n}^{*}\left(\theta \right),$$

where $\Phi_{k}^{*}\left(\theta \right)={\left(I-\Phi_{0}\left(\theta \right)\right)}^{-1}\Phi_{k}\left(\theta \right)$, and $\Delta M_{n}^{*}\left(\theta \right)={\left(I-\Phi_{0}\left(\theta \right)\right)}^{-1}\Delta M_{n}\left(\theta \right)$ is a zero-mean, cross-correlated white noise sequence satisfying

$$\begin{array}{cc} E[\Delta M_{n}^{*}\left(\theta \right){\Delta M_{n^{\prime }}^{*}\left(\theta \right)}^{T}]\; & =\delta_{nn^{\prime }}{\left(I-\Phi_{0}\left(\theta \right)\right)}^{-1}\mathrm{diag}\left(\lambda \right){\left(I-\Phi_{0}{\left(\theta \right)}^{T}\right)}^{-1}\\ \; & =:\delta_{nn^{\prime }}\Lambda^{*}\left(\theta \right),\;n,n^{\prime }\in Z. \end{array}$$

Based on this representation, we define a loss function in quadratic form, whose minimizer yields an estimator of the parameter vector:

(19) $$\begin{array}{c} L_{n}\left(\theta \right)=\sum_{k=1}^{n}{\left(Y_{k}-\sum_{j=1}^{k-1}\Phi_{j}^{*}\left(\theta \right)Y_{k-j}\right)}^{T}{\Lambda^{*}}^{-1}\left(\theta \right)\left(Y_{k}-\sum_{j=1}^{k-1}\Phi_{j}^{*}\left(\theta \right)Y_{k-j}\right)+n\log\left|\Lambda^{*}\left(\theta \right)\right|, \end{array}$$

where $Y_{k}=X_{k}-\lambda$. In practice, the unknown mean vector λ is replaced by the empirical mean $\hat{\lambda }=n^{-1}\sum_{k=1}^{n}X_{k}$. The loss function then simplifies to

(20) $$\begin{array}{c} L_{n}\left(\theta \right)=\sum_{k=1}^{n}{\left(Y_{k}-\sum_{j=0}^{k-1}\Phi_{j}\left(\theta \right)Y_{k-j}\right)}^{T}\mathrm{diag}{\left(\hat{\lambda }\right)}^{-1}\left(Y_{k}-\sum_{j=0}^{k-1}\Phi_{j}\left(\theta \right)Y_{k-j}\right)-2n\log\left|I-\Phi_{0}\left(\theta \right)\right|, \end{array}$$

where the constant term $\log\mathrm{diag}(\hat{\lambda })$ has been omitted. The first term on the right-hand side of (20) corresponds to a weighted quadratic loss, while the second term serves as a regularization component that discourages trivial solutions of the form $\Phi_{k}\left(\theta \right)=\delta_{k0}I$. The optimal parameter estimate is obtained by minimizing the loss function with respect to θ, which can be efficiently carried out using a gradient-based optimization algorithm.

The estimation procedure is summarized as shown in Algorithm 1.
**Algorithm 1** Estimation procedure for Hawkes processes1:Given the observed bin-count sequence $\left\{X_{1},\ldots ,X_{n}\right\}$, compute the empirical mean $\hat{\lambda }=n^{-1}\sum_{k=1}^{n}X_{k}$ and center the data as $Y_{k}=X_{k}-\hat{\lambda }$ for k=1,…,n.2:Determine the parameter estimate $\hat{\theta }$ by minimizing the loss function (20).3:Using the estimated parameter vector $\hat{\theta }$, compute the estimate of the baseline intensity as$$\hat{\mu }=\left(I-\hat{A}\right)\hat{\lambda }/\Delta ,$$
where $\hat{A}=\sum_{n=0}^{\infty }\Phi_{n}\left(\hat{\theta }\right)$ denotes the estimated branching ratio matrix.

## 4. Numerical Experiments

### 4.1. Assessment of Second-Order Characteristics

As established in Corollary 1, the coarse-grained Hawkes process asymptotically approximates the spectral density matrix of the binned Hawkes process as the bin size Δt→0. In this section, we conduct numerical investigations to evaluate the validity and robustness of this approximation for increasing values of Δt. To this end, we consider a bivariate Hawkes process (d=2), where each component of the excitation kernel matrix is defined as

$$\phi_{ij}\left(t\right)=\alpha_{ij}g_{ij}\left(t\right),\;t\geq 0,$$

with $g_{ij}\left(t\right)$ denoting a normalized kernel satisfying $\int_{0}^{\infty }g_{ij}\left(t\right)dt=1$. The coefficient $\alpha_{ij}$ specifies the branching ratio from component *j* to component *i*, and $g_{ij}\left(t\right)$ characterizes the distribution of waiting times for event excitation. We specifically focus on a symmetric bivariate Hawkes process where the parameters satisfy $\mu_{1}=\mu_{2}=\mu$, $g_{ij}\left(t\right)=g\left(t\right)$ (1≤i,j≤2), and the excitation kernel matrix takes the form

$$\left(\begin{array}{cc} \alpha_{11} & \alpha_{12}\\ \alpha_{21} & \alpha_{22} \end{array}\right)=\left(\begin{array}{cc} \alpha^{\left(s\right)} & \alpha^{\left(c\right)}\\ \alpha^{\left(c\right)} & \alpha^{\left(s\right)} \end{array}\right).$$

The stationarity condition for the process holds if $\alpha^{\left(s\right)}+\alpha^{\left(c\right)}<1$. Under this constraint, the second-order statistical structure of the symmetric Hawkes process is described by the power spectral density (PSD) of each component (the diagonal elements of the spectral density matrix) and the cross-spectral density (CSD) between them (the off-diagonal elements).

**Figure 1:**
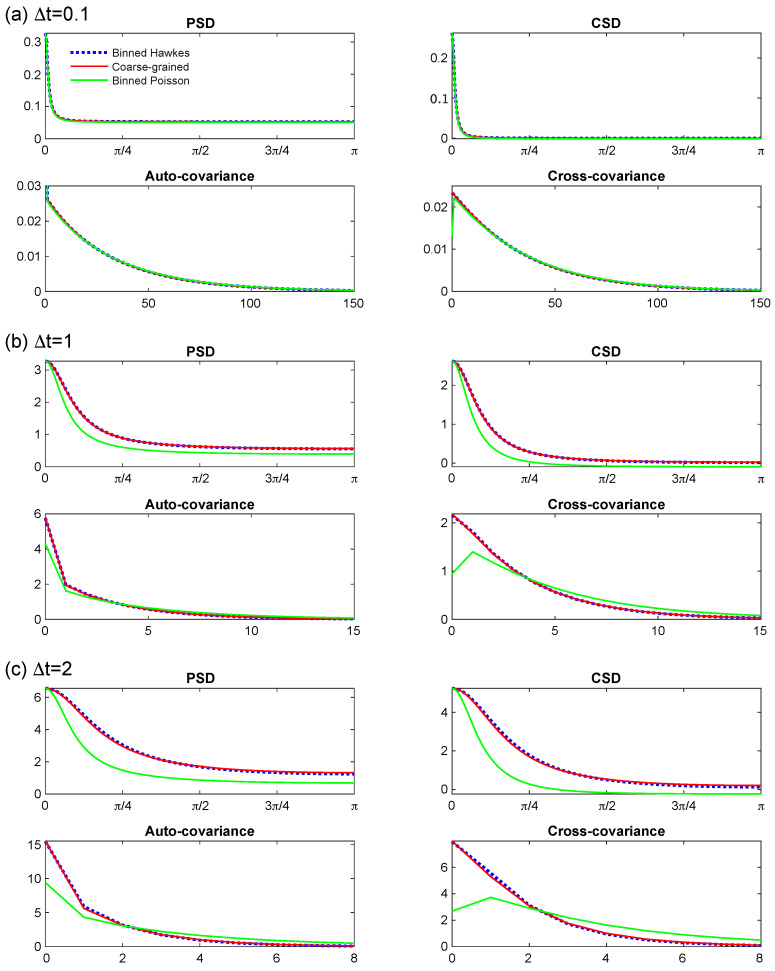
Power spectral density (PSD), cross-spectral density (CSD), auto-covariance, and cross-covariance functions for the three processes at bin sizes Δt=0.1 (**a**), 1 (**b**), and 2 (**c**). The blue dotted line corresponds to the binned Hawkes process, while the red and green lines depict the coarse-grained Hawkes process and the binned Poisson approximation, respectively. The parameters of the Hawkes process are set as μ=1, $\alpha^{\left(s\right)}=0.4$, $\alpha^{\left(c\right)}=0.3$, and β=1. The coarse-grained Hawkes process provides a close approximation to the binned Hawkes process, whereas the binned Poisson approximation exhibits noticeable degradation for Δt=1 and 2.

We illustrate our findings using an exponential kernel defined as

$$g\left(t\right)=\beta e^{-\beta t},\;t\geq 0,$$

where $\beta^{-1}$ denotes the expected waiting time before excitation. Figure 1 presents the PSD and CSD, along with the auto- and cross-covariance functions, of the binned Hawkes process with parameters $\alpha^{\left(s\right)}=0.4$, $\alpha^{\left(c\right)}=0.3$, and β=1, for bin sizes Δt=0.1 (a), 1 (b), and 2 (c). These are depicted using blue dotted lines.

The associated coarse-grained Hawkes process is constructed using the coarse-grained kernel derived from the exponential function:

$$g_{k}=\left\{\begin{array}{cc} 1-(1-e^{-\beta \Delta t})/\beta \Delta t, & k=0,\\ \left(e^{\beta \Delta t}+e^{-\beta \Delta t}-2\right)e^{-\beta k\Delta t}/\beta \Delta t, & k=1,2,\ldots \end{array}\right.$$

In the same figures, the four second-order statistics of the coarse-grained process are represented by red lines, while those corresponding to the binned Poisson approximation are shown in green.

From these comparisons, we observe that for small bin sizes ($\Delta t=0.1<\beta^{-1}$), both the coarse-grained Hawkes process and the binned Poisson approximation closely reproduce the second-order behavior of the binned Hawkes process (Figure 1a). However, for Δt=1, which is comparable to the mean waiting time, the Poisson approximation significantly deteriorates (Figure 1b), whereas the coarse-grained Hawkes process continues to provide a high-fidelity approximation. Even for a larger bin size Δt=2, exceeding the characteristic time scale $\beta^{-1}$, the coarse-grained model remains accurate (Figure 1c).

To quantitatively assess the fidelity of the approximations, we introduce a divergence measure based on the log-determinant of the spectral density matrices. Specifically, let $F_{\Delta t}^{\left(••\right)}\left(\omega \right)$ denote the spectral density matrix of the approximating process, where ‘••’ indicates either the coarse-grained (cg) or Poisson (po) approximation. Then, the information loss relative to the binned Hawkes process $F_{\Delta t}^{\left(hw\right)}\left(\omega \right)$ is defined as

$$\Delta h^{\left(••\right)}=\frac{1}{4\pi }\int_{-\pi }^{\pi }\log\frac{|F_{\Delta t}^{\left(hw\right)}\left(\omega \right)|}{|F_{\Delta t}^{\left(••\right)}\left(\omega \right)|}d\omega ,$$

which corresponds to the gap in maximum entropy rates under spectral constraints.

**Figure 2:**
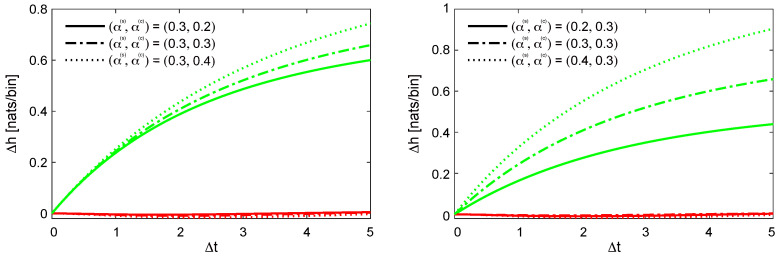
Information loss as a function of Δt for the coarse-grained Hawkes process (red line) and the binned Poisson approximation (green line). The parameters are set to μ=1 and β=1. The information loss associated with the binned Poisson approximation increases with larger values of Δt, $\alpha^{\left(s\right)}$, and $\alpha^{\left(c\right)}$, whereas the information loss incurred by the coarse-grained Hawkes process remains negligible across all configurations.

Figure 2 plots the information loss for both approximations as a function of Δt, across varying values of $\alpha^{\left(s\right)}$ and $\alpha^{\left(c\right)}$. The results clearly indicate that the coarse-grained Hawkes process incurs minimal information loss across a wide range of parameter settings. In contrast, the accuracy of the binned Poisson approximation degrades with increasing Δt, with the loss exacerbated further as $\alpha^{\left(s\right)}$ and $\alpha^{\left(c\right)}$ increase.

**Figure A1:**
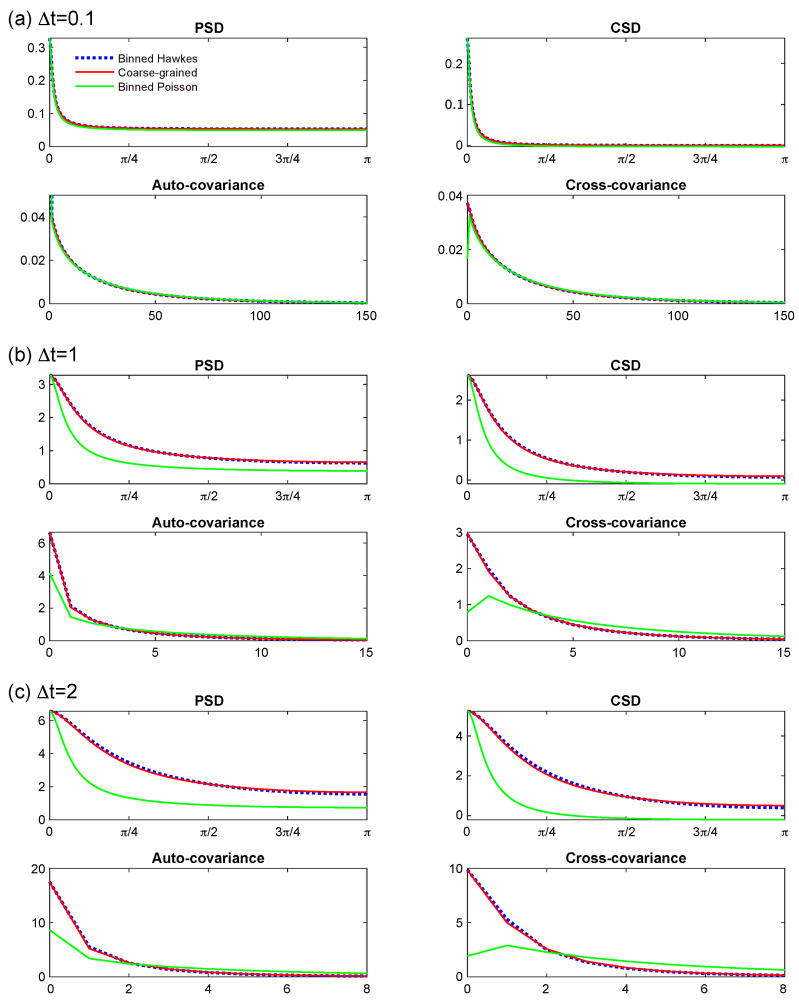
Same as Figure 1, but for the power-law kernel. Parameters of the Hawkes process are set to μ=1, $\alpha^{\left(s\right)}=0.4$, $\alpha^{\left(c\right)}=0.3$, β=1, and γ=2. (**a**) Δt = 0.1, (**b**) Δt = 1, (**c**) Δt = 2.

**Figure A2:**
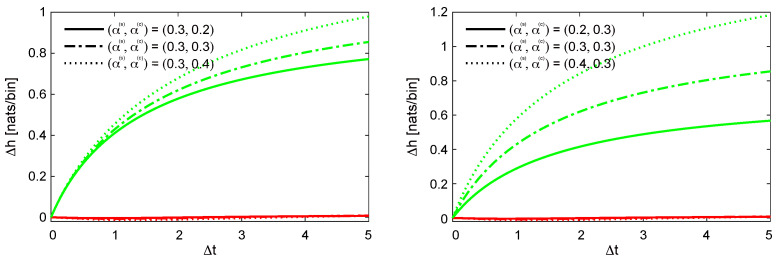
Same as Figure 2, but for the power-law kernel. Parameters are set to μ=1, β=1, and γ=2.

We additionally examined the case where the excitation kernel follows a power-law distribution instead of an exponential decay. The qualitative behavior remained consistent (see Appendix C; Figure A1 and Figure A2). These findings collectively demonstrate that the coarse-grained Hawkes process offers a substantially improved approximation of the second-order dynamics of the binned Hawkes process, particularly for larger bin widths.

### 4.2. Parameter Estimation

We now investigate the efficacy of the proposed estimation method in inferring the parameters of a Hawkes process from bin-count data. Specifically, we consider an asymmetric bivariate Hawkes process with exponential excitation kernels given by $g_{ij}\left(t\right)=\beta_{ij}e^{-\beta_{ij}t}$ (1≤i,j≤2). The parameters of the Hawkes process are set as follows:

$$\left(\begin{array}{c} \mu_{1}\\ \mu_{2} \end{array}\right)=\left(\begin{array}{c} 1\\ 1 \end{array}\right),\;\left(\begin{array}{cc} \alpha_{11} & \alpha_{12}\\ \alpha_{21} & \alpha_{22} \end{array}\right)=\left(\begin{array}{cc} 0.4 & 0.5\\ 0.3 & 0.2 \end{array}\right),\;\left(\begin{array}{cc} \beta_{11} & \beta_{12}\\ \beta_{21} & \beta_{22} \end{array}\right)=\left(\begin{array}{cc} 0.5 & 0.7\\ 0.3 & 1.0 \end{array}\right).$$

The numerical experiments were conducted as follows. First, realizations of the Hawkes process were generated over the interval [0,T] and subsequently discretized into bin-count sequences using bin size Δt. The ten model parameters ${\left\{\mu_{i},\alpha_{ij},\beta_{ij}\right\}}_{1\leq i,j\leq 2}$ were then estimated from these sequences. Parameter estimation was performed by minimizing the loss function (20) using the quasi-Newton method (BFGS), with finite difference approximation employed for gradient evaluation.

To assess the performance of our proposed method, we compare it against three established approaches. The first is the MC-EM algorithm proposed in [20], for which we employed the publicly available implementation [25]. To ensure a fair comparison, we adopted the tuning parameters specified in [20], setting the number of Monte Carlo samples to 10. The second method is maximum likelihood estimation (MLE) applied to the binned Poisson approximation, with the MLE computed using the quasi-Newton method (BFGS). The third method involves conditional least squares estimation for the INAR(*p*) process, as introduced in [18]. Since the INAR(*p*) framework yields nonparametric estimates of the excitation kernels, we obtained the corresponding parametric kernel parameters by fitting an exponential function to the nonparametric estimates [19,20].

**Figure 3:**
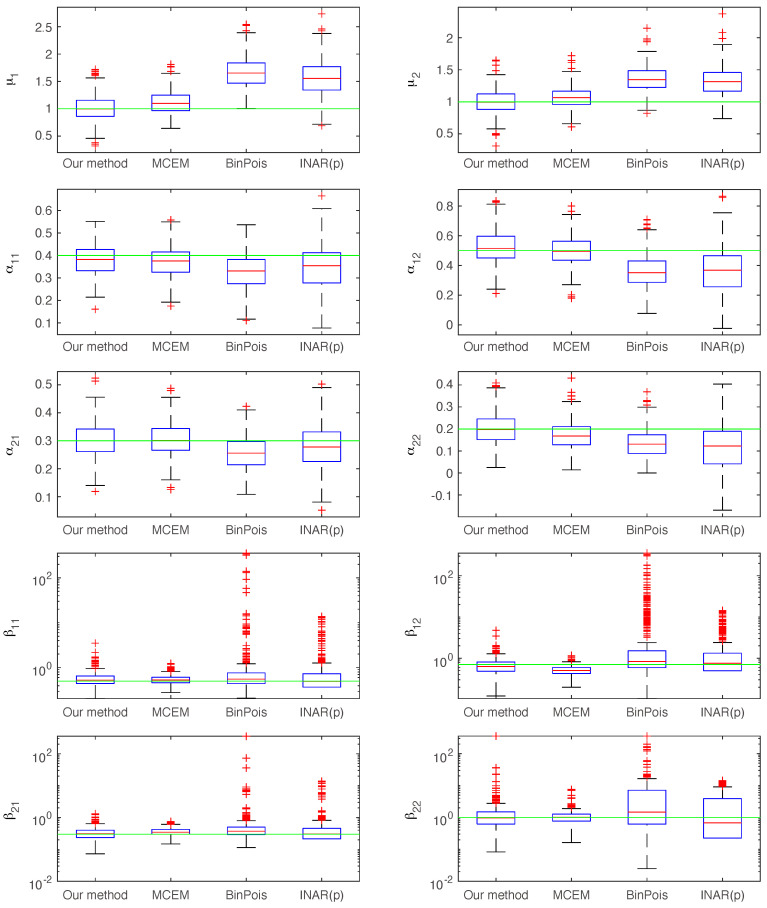
Boxplots of the estimated values for each of the ten model parameters. The green solid line indicates the ground truth values. Note that the INAR(*p*) method may yield negative values, which are omitted when log-scaled axes are used.

Figure 3 presents boxplots of the estimated values for each of the ten parameters across 500 simulated realizations of the binned Hawkes process, using T=1000 and Δt=2. It is apparent that both the binned Poisson MLE method and the INAR(*p*) method produce significantly biased estimates, which is expected given that these approaches disregard excitation effects within each bin. Furthermore, we observe a substantial number of outliers in the estimates of $\beta_{ij}$ across all four methods, indicating that estimation of the kernel scales exhibits higher variance than estimation of the baseline intensities or branching ratios. For instance, our method yielded estimates of $\beta_{22}$ that deviated by a factor of 100 from the true value in 16 out of the 500 trials, highlighting the challenges in accurately estimating $\beta_{ij}$ when the bin size is large relative to the kernel time scale.

**Figure 4:**
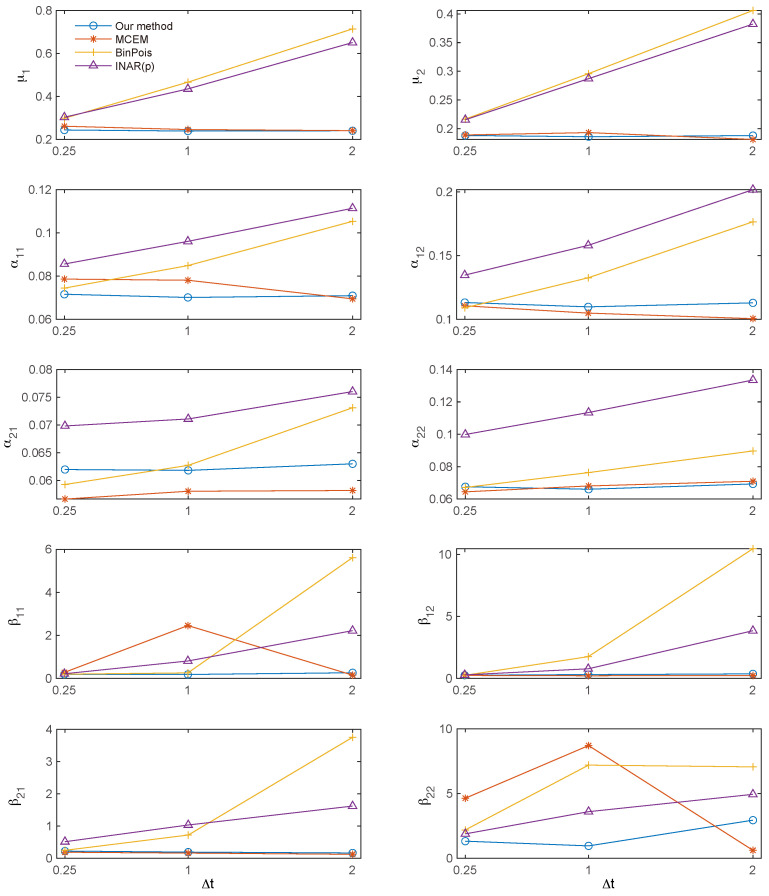
Root mean squared error (RMSE) of the parameter estimates. Overall, the RMSE of the proposed method is comparable to that of the MC-EM algorithm, and significantly lower than that of the binned Poisson approximation and the INAR(*p*) method.

Figure 4 displays the root mean squared error (RMSE) of the parameter estimates across various bin sizes. For the RMSE of ${\hat{\beta }}_{ij}$, extreme outliers with values exceeding 100 were excluded to mitigate their undue influence. Overall, the proposed method demonstrates superior performance compared to both the binned Poisson MLE and the INAR(*p*) approach, and achieves accuracy comparable to the MC-EM algorithm. Additionally, both the MC-EM and the proposed methods consistently maintain low RMSE values across different bin sizes, whereas the RMSEs for the binned Poisson MLE and the INAR(*p*) approach increase with larger bin sizes. A similar trend is observed for ${\hat{\beta }}_{ij}$, albeit with more fluctuation due to outliers.

**Figure 5:**
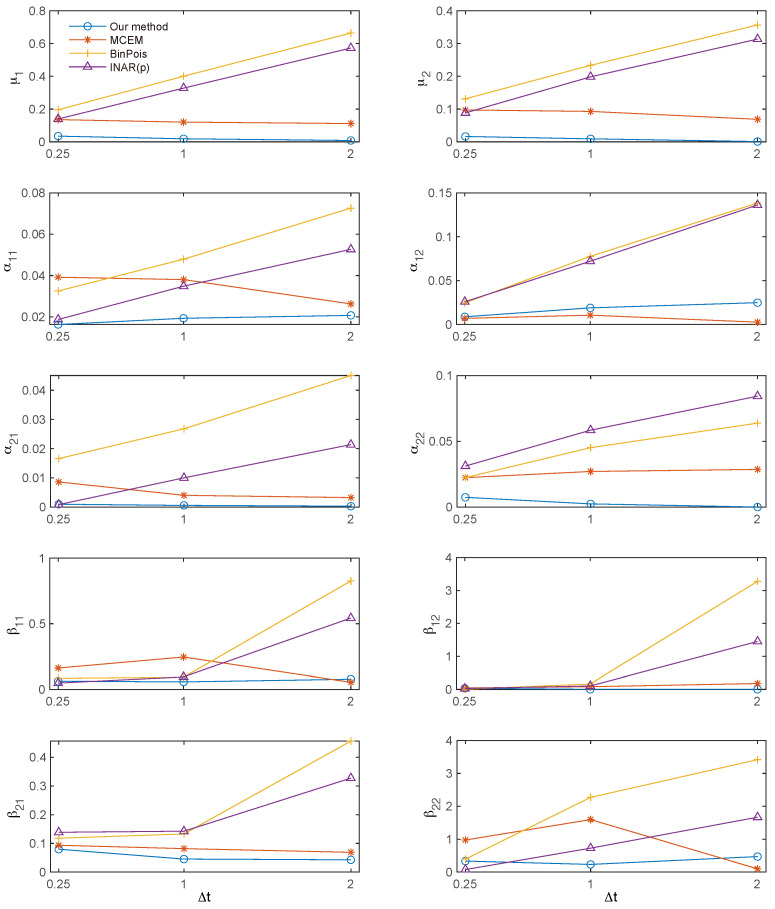
Bias in the estimated parameters. The proposed method consistently achieves the lowest bias among the four methods, except for a few isolated cases.

**Figure 6:**
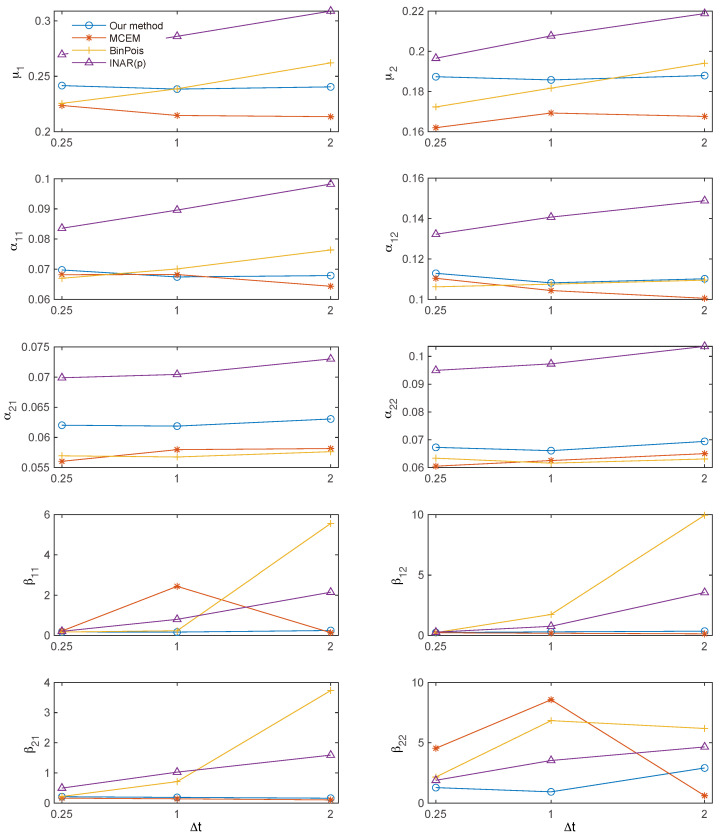
Standard deviation (STD) in the parameter estimates. The MC-EM algorithm generally attains the lowest standard deviation among the four methods.

Figure 5 and Figure 6 illustrate, respectively, the bias and standard deviation components of the RMSE for each of the ten parameters. The proposed method yields the lowest bias among all methods, with only a few exceptions (Figure 5); meanwhile, the MC-EM algorithm attains the lowest standard deviation (Figure 6).

**Figure A3:**
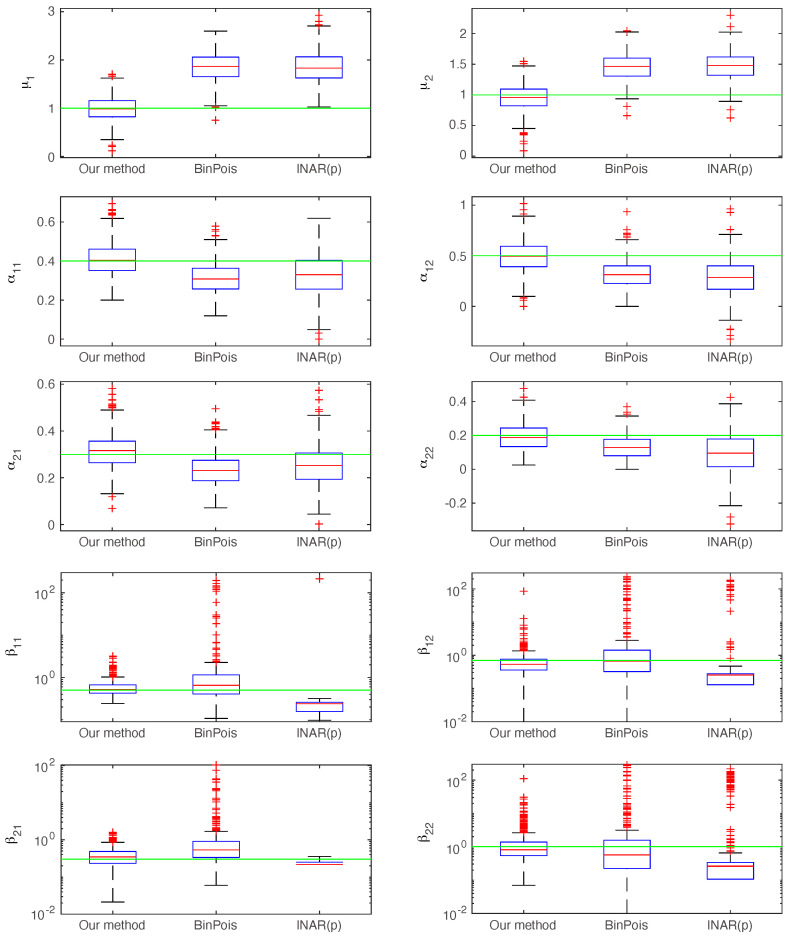
Boxplots of the estimated values for each of the ten model parameters. Same as Figure 3, but based on power-law kernels. The power-law exponent is set to γ=2, with all other parameters identical to those used in the exponential kernel scenario.

**Figure A4:**
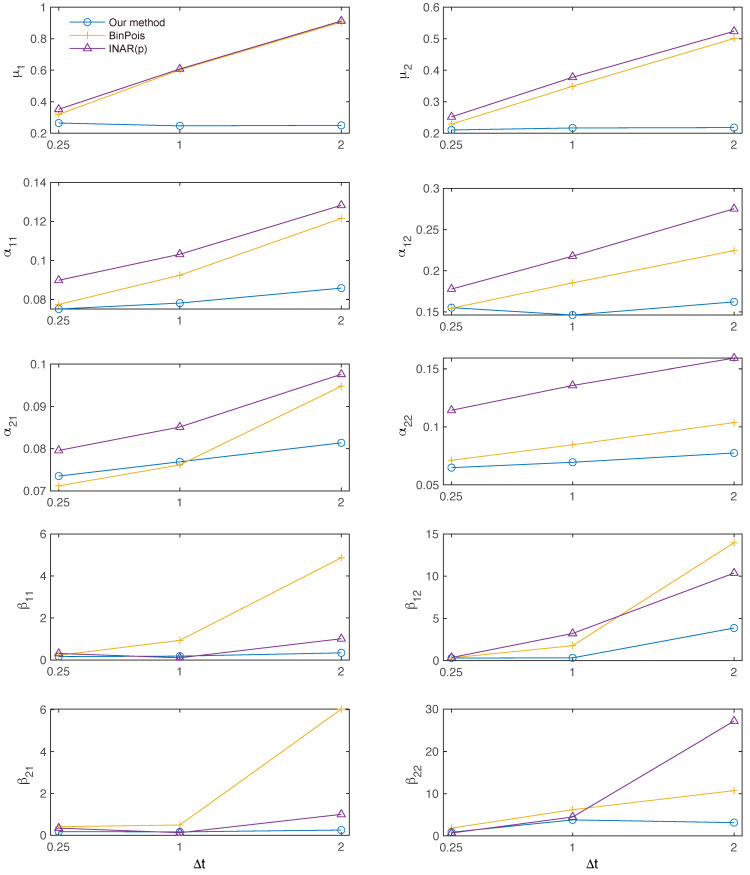
Root mean squared error (RMSE) of the parameter estimates. Same as Figure 4, but evaluated using power-law kernels.

**Figure A5:**
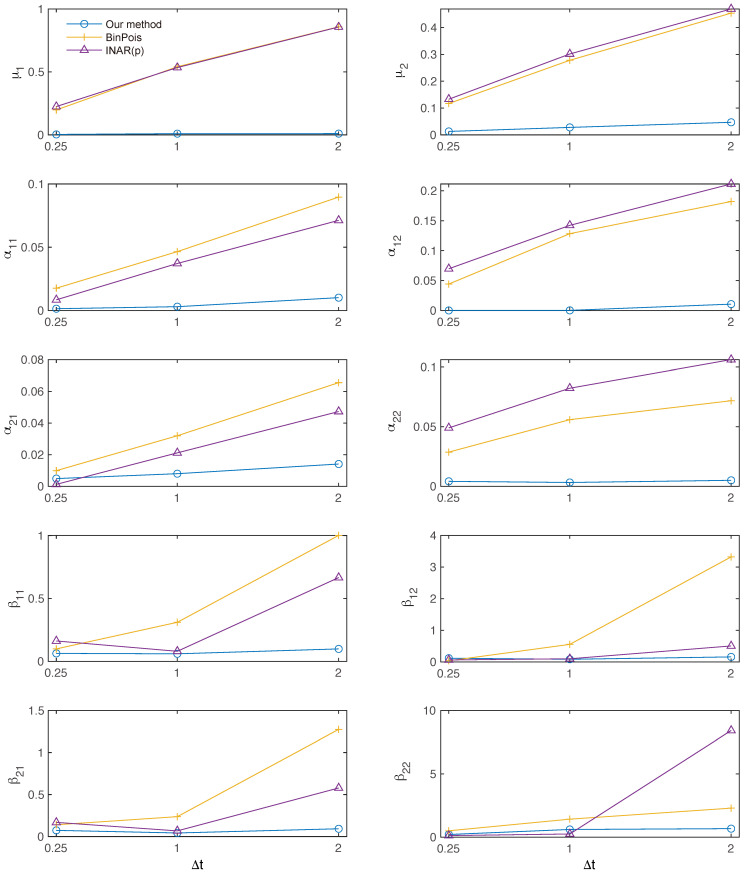
Bias in the estimated parameters. Same as Figure 5, but for the power-law kernel setting.

**Figure A6:**
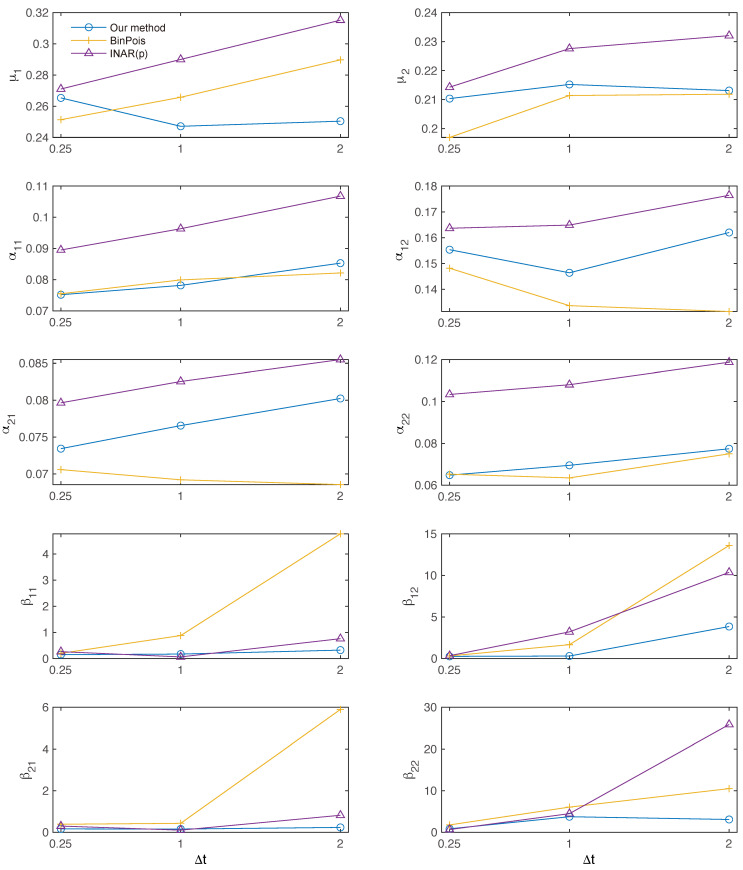
Standard deviation (STD) of the parameter estimates. Same as Figure 6, but for the power-law kernel case.

We further investigated the case in which the excitation kernel follows a power-law distribution rather than an exponential decay, thereby testing a different (non-memoryless) kernel. Our results confirm that the proposed method remains effective in this setting (Appendix C; Figure A3, Figure A4, Figure A5 and Figure A6).

In conclusion, the proposed method matches the performance of the MC-EM algorithm while outperforming both the binned Poisson MLE and the INAR(*p*) method, particularly in terms of bias reduction. It provides robust and stable estimates for the baseline intensities $\mu_{i}$ and the branching ratios $\alpha_{ij}$, with estimation accuracy largely unaffected by bin size. Accurate estimation of the kernel scales $\beta_{ij}$ is feasible when the bin size is smaller than the characteristic kernel scale, but becomes unreliable as the bin size increases.

### 4.3. Choice of Parametric Form of Excitation Kernel

**Figure 7:**
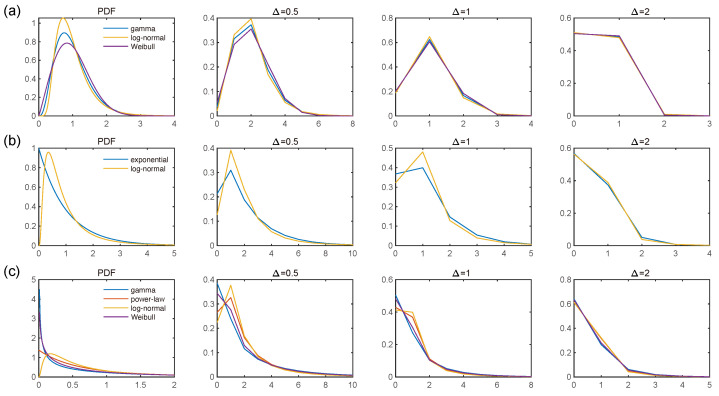
PDFs and their coarse-grained counterparts for Δt=0.5, 1, and 2. The mean of all PDFs is 1, and the standard deviation (STD) is (**a**) 0.5, (**b**) 1, and (**c**) 1.5. Note that the gamma and Weibull distributions converge to the exponential distribution when STD=1. Additionally, the power-law distribution is not defined for STD≤1.

To investigate whether the statistical properties of the coarse-grained Hawkes process are influenced by the specific choice of parametric kernel function, we considered four probability density functions (PDFs): gamma, power-law, log-normal, and Weibull. Figure 7 illustrates the PDFs of these four distributions, all of which share a common mean and standard deviation, with their coarse-grained counterparts. It is evident that as the bin size Δt increases, the coarse-grained kernels converge and become indistinguishable from one another, as the detailed shape of the distributions is averaged out. This observation suggests that our method is robust to the parametric form of the excitation kernel when the bin size is large relative to the kernel timescale.

## 5. Discussion

In this study, we introduced the coarse-grained Hawkes process as an analytical approximation to the binned Hawkes process. Unlike conventional discretization techniques, the proposed framework incorporates a coarse-grained excitation kernel that systematically accounts for intra-bin excitations. Consequently, the coarse-grained Hawkes process faithfully reproduces the second-order statistical properties of the binned Hawkes process, even when the bin size exceeds the characteristic timescale of the excitation kernel. Moreover, we demonstrated that the proposed approach enables stable estimation of Hawkes process parameters from bin-count data. In particular, both the branching ratios and baseline intensities can be reliably inferred, irrespective of the temporal resolution of the bin-count sequences.

A central distinction between our approach and the Monte Carlo Expectation-Maximization (MC-EM) algorithm lies in the treatment of latent event times within the bin-count data. Whereas the MC-EM method necessitates Monte Carlo sampling from the conditional distribution of unobserved events, resulting in considerable computational burden, our method employs a parsimonious assumption that events are uniformly distributed within each bin. This assumption facilitates the analytical derivation of approximate conditional expectations. Despite its simplicity, the proposed method achieves estimation accuracy on par with that of the MC-EM algorithm while offering substantial computational advantages.

An additional strength of our approach is its robustness to the parametric form of the excitation kernel as the bin size increases. As the detailed shape of the kernel becomes averaged out, the parametric specification becomes largely irrelevant. Consequently, for large bin sizes relative to the kernel timescale, our method remains effective irrespective of the precise functional form of the excitation kernel.

We further highlight a theoretical connection between our estimation framework and the spectral method, previously validated in the univariate setting [22]. Applying the Fourier transform, the loss function in Equation (19) asymptotically approximates a spectral likelihood for large *n*:

$$L_{n}\left(\theta \right)\approx \sum_{m=1}^{n}\left({\hat{X}}_{m}^{*}F_{\Delta t}^{\left(cg\right)}{\left(\omega_{m}\right)}^{-1}{\hat{X}}_{m}+\log\left|F_{\Delta t}^{\left(cg\right)}\left(\omega_{m}\right)\right|\right),$$

where $\omega_{m}=2\pi m/n$,

$${\hat{X}}_{m}=\frac{1}{\sqrt{2\pi n}}\sum_{k=1}^{n}\left({\tilde{X}}_{k}-\lambda \right)e^{-2\pi ikm/n},$$

and ${\hat{X}}_{m}^{*}$ denotes the Hermitian transpose. Notably, the spectral likelihood for the multivariate binned Hawkes process can be obtained by replacing $F_{\Delta t}^{\left(cg\right)}\left(\omega_{m}\right)$ with the spectral density matrix of the binned Hawkes process.

Although our analysis focused on temporal Hawkes processes, the proposed modeling framework readily extends to space-time Hawkes processes. By discretizing both the temporal and spatial domains and counting the number of events in each bin, one obtains multivariate bin-count sequences to which the coarse-grained Hawkes process can be applied. In this extension, the coarse-graining procedure must be conducted in both time and space.

Finally, we emphasize the potential extension of the proposed framework to nonstationary time series. Owing to its formulation in the time domain, the coarse-grained Hawkes process is amenable to integration within state-space modeling paradigms [26,27], offering a promising direction for modeling nonstationary dynamics. We propose this as a compelling avenue for future research.

## Data Availability

The original contributions presented in this study are included in the article. Further inquiries can be directed to the corresponding author.

## Appendix A. Proofs

### Appendix A.1. Proof of Lemma 1

It follows from Equations (6) and (7) that

$$\begin{array}{cc} \sum_{k=0}^{\infty }\phi_{k}\; & =\lim_{n\to \infty }\left\{\xi_{0}+\left(\xi_{1}-\xi_{0}\right)+\ldots +\left(\xi_{n}-\xi_{n-1}\right)\right\}=\lim_{n\to \infty }\xi_{n}\\ \; & =\lim_{n\to \infty }\frac{1}{\Delta t}\int_{n\Delta t}^{(n+1)\Delta t}\left(\int_{0}^{t}\phi \left(u\right)du\right)dt=\lim_{n\to \infty }\frac{1}{\Delta t}\int_{0}^{\Delta t}\left(\int_{0}^{n\Delta t+s}\phi \left(u\right)du\right)ds. \end{array}$$

Since $\int_{0}^{n\Delta t+s}\phi \left(u\right)du$ is nonnegative and monotonically nondecreasing with respect to *n*, we may invoke the dominated convergence theorem to interchange the limit and the integral, yielding

$$\begin{array}{c} \sum_{k=0}^{\infty }\phi_{k}=\frac{1}{\Delta t}\int_{0}^{\Delta t}\left(\lim_{n\to \infty }\int_{0}^{n\Delta t+s}\phi \left(u\right)du\right)ds=\frac{1}{\Delta t}\int_{0}^{\Delta t}\left(\int_{0}^{\infty }\phi \left(u\right)du\right)ds=\alpha . \end{array}$$

Furthermore, since $\int_{0}^{t}\phi \left(u\right)du$ is continuous, nonnegative, and monotonically nondecreasing in *t*, we obtain

$$\begin{array}{c} \phi_{0}=\frac{1}{\Delta t}\int_{0}^{\Delta t}\left(\int_{0}^{t}\phi \left(u\right)du\right)dt<\frac{1}{\Delta t}\int_{0}^{\Delta t}\left(\int_{0}^{\infty }\phi \left(u\right)du\right)dt=\alpha . \end{array}$$

### Appendix A.2. Derivation of (12) and (13)

From Equations (8) and (10), we have

$$\begin{array}{cc} E[{\tilde{X}}_{n}|H_{n-1}]\; & =E\left[\mu \Delta t+\sum_{k\leq n}\Phi_{n-k}{\tilde{X}}_{k}|H_{n-1}\right]\\ \; & =\mu \Delta t+\sum_{k\leq n-1}\Phi_{n-k}{\tilde{X}}_{k}+\Phi_{0}E\left[{\tilde{X}}_{n}|H_{n-1}\right]. \end{array}$$

$$∴\;\left(I-\Phi_{0}\right)E\left[{\tilde{X}}_{n}|H_{n-1}\right]=\mu \Delta t+\sum_{k\leq n-1}\Phi_{n-k}{\tilde{X}}_{k}.$$

Since the spectral radius of $\Phi_{0}$ is strictly less than one, the matrix $\left(I-\Phi_{0}\right)$ is invertible, and all entries of $E[{\tilde{X}}_{n}|H_{n-1}]$ are nonnegative. Hence, the conditional mean vector of ${\tilde{X}}_{n}$ is given by expression (12).

To derive (13), observe that

$$\begin{array}{c} {\tilde{X}}_{n}-\lambda_{n}^{*}={\left(I-\Phi_{0}\right)}^{-1}\left(\left(I-\Phi_{0}\right){\tilde{X}}_{n}-\mu \Delta t-\sum_{k\leq n-1}\Phi_{n-k}{\tilde{X}}_{k}\right)={\left(I-\Phi_{0}\right)}^{-1}\Delta M_{n}. \end{array}$$

Therefore,

$$\begin{array}{cc} \mathrm{Var}({\tilde{X}}_{n}|H_{n-1})\; & =E\left[\left({\tilde{X}}_{n}-\lambda_{n}^{*}\right){\left({\tilde{X}}_{n}-\lambda_{n}^{*}\right)}^{T}|H_{n-1}\right]\\ \; & ={\left(I-\Phi_{0}\right)}^{-1}E\left[\Delta M_{n}\Delta M_{n}^{T}|H_{n-1}\right]{\left(I-\Phi_{0}^{T}\right)}^{-1}\\ \; & ={\left(I-\Phi_{0}\right)}^{-1}\mathrm{diag}\left(E\left[\lambda_{n}|H_{n-1}\right]\right){\left(I-\Phi_{0}^{T}\right)}^{-1}, \end{array}$$

where the last equality follows from Equation (11). Finally, since $E\left[\lambda_{n}|H_{n-1}\right]=\lambda_{n}^{*}$ by (10), we obtain Equation (13).

### Appendix A.3. Proof of Lemma 2

From Equation (10), it follows that

$$E\left[\Delta M_{n}\right]=E\left[E[\Delta M_{n}|H_{n-1}]\right]=0.$$

To derive Equation (15), observe that for $n<n^{\prime }$ (and, by symmetry, for $n\neq n^{\prime }$),

$$E\left[\Delta M_{n}\Delta M_{n^{\prime }}^{T}\right]=E\left[\Delta M_{n}E\left[\Delta M_{n^{\prime }}^{T}|H_{n^{\prime }-1}\right]\right]=0.$$

Moreover, applying Equation (11), we obtain

$$\begin{array}{c} E\left[\Delta M_{n}\Delta M_{n}^{T}\right]=E\left[E[\Delta M_{n}\Delta M_{n}^{T}|H_{n-1}]\right]=E\left[\mathrm{diag}(E\left[\lambda_{n}|H_{n-1}\right])\right]=\mathrm{diag}\left(\lambda \right), \end{array}$$

which completes the proof.

### Appendix A.4. Ar(∞) and MA(∞) Representations

Using Equations (9) and (14), the rate Equation (8) can be reformulated as

(A1) $${\tilde{X}}_{n}-\lambda =\sum_{k=0}^{\infty }\Phi_{k}\left({\tilde{X}}_{n-k}-\lambda \right)+\Delta M_{n},$$

$$∴\;\left(I-\Phi_{0}\right)\left({\tilde{X}}_{n}-\lambda \right)=\sum_{k=1}^{\infty }\Phi_{k}\left({\tilde{X}}_{n-k}-\lambda \right)+\Delta M_{n}.$$

Under the stationarity condition, the inverse ${\left(I-\Phi_{0}\right)}^{-1}$ exists, yielding the autoregressive representation

$${\tilde{X}}_{n}-\lambda =\sum_{k=1}^{\infty }\Phi_{k}^{*}\left({\tilde{X}}_{n-k}-\lambda \right)+\Delta M_{n}^{*},$$

where $\Phi_{k}^{*}={\left(I-\Phi_{0}\right)}^{-1}\Phi_{k}$ and $\Delta M_{n}^{*}={\left(I-\Phi_{0}\right)}^{-1}\Delta M_{n}$.

To obtain a moving average representation, we apply the formal *z*-transform to both sides of Equation (A1):

$$\hat{Y}\left(z\right)=\hat{\Phi }\left(z\right)\hat{Y}\left(z\right)+\Delta \hat{M}\left(z\right),$$

where $\hat{Y}\left(z\right)=\sum_{n=-\infty }^{\infty }\left({\tilde{X}}_{n}-\lambda \right)z^{-n}$, $\hat{\Phi }\left(z\right)=\sum_{n=-\infty }^{\infty }\Phi_{n}z^{-n}$ and $\Delta \hat{M}\left(z\right)=\sum_{n=-\infty }^{\infty }\Delta M_{n}z^{-n}$. Solving for $\hat{Y}\left(z\right)$ yields

$$\hat{Y}\left(z\right)=\hat{\Psi }\left(z\right)\Delta \hat{M}\left(z\right),$$

where the transfer function is defined by

(A2) $$\hat{\Psi }\left(z\right)={\left(I-\hat{\Phi }\left(z\right)\right)}^{-1}=\sum_{j=0}^{\infty }{\hat{\Phi }}^{j}\left(z\right).$$

Applying the inverse *z*-transform then gives the MA(*∞*) process:

$${\tilde{X}}_{n}-\lambda =\sum_{k=0}^{\infty }\Psi_{k}\Delta M_{n-k},$$

where $\Psi_{k}=\sum_{j=0}^{\infty }{\left(\Phi^{\left(*j\right)}\right)}_{k}$. Furthermore, utilizing Equation (A2), and noting that $\hat{\Psi }\left(1\right)=\sum_{n=0}^{\infty }\Psi_{n}$ and $\hat{\Phi }\left(1\right)=A$, the branching ratio matrix can be expressed in terms of $\Psi_{n}$ as $A=I-{\left(\sum_{n=0}^{\infty }\Psi_{n}\right)}^{-1}$.

### Appendix A.5. Proof of Theorem 1

Using Equations (15) and (16), the autocovariance matrix of $\left\{{\tilde{X}}_{n}\right\}$ is given by

$$\begin{array}{cc} \mathrm{Cov}({\tilde{X}}_{n},{\tilde{X}}_{n+j})\; & =E[\left({\tilde{X}}_{n}-\lambda \right){\left({\tilde{X}}_{n+j}-\lambda \right)}^{T}]\\ \; & =E\left[\left(\sum_{k=0}^{\infty }\Psi_{k}\Delta M_{n-k}\right){\left(\sum_{l=0}^{\infty }\Psi_{l}\Delta M_{n+j-l}\right)}^{T}\right]\\ \; & =\sum_{k=0}^{\infty }\sum_{l=0}^{\infty }\Psi_{k}E\left[\Delta M_{n-k}\Delta M_{n+j-l}^{T}\right]\Psi_{l}^{T}\\ \; & =\sum_{k=0}^{\infty }\sum_{l=0}^{\infty }\Psi_{k}\mathrm{diag}\left(\lambda \right)\delta_{k,l-j}\Psi_{l}^{T}\\ \; & =\sum_{l=0}^{\infty }\Psi_{l-j}\mathrm{diag}\left(\lambda \right)\Psi_{l}^{T}, \end{array}$$

which completes the proof.

### Appendix A.6. Proof of Theorem 2

We provide a proof of Theorem 2 in the univariate setting. The extension to the multivariate case follows by applying the same reasoning component-wise to each element of the spectral density matrix. To begin, we establish the following approximation result for the Fourier transforms of the excitation kernel and its coarse-grained counterpart.

**Lemma** **A1.**
*Let $\tilde{\phi }\left(\nu \right)=\int_{0}^{\infty }\phi \left(t\right)e^{-i\nu t}dt$ denote the continuous-time Fourier transform of the excitation kernel, and let $\hat{\phi }\left(\omega \right)=\sum_{k=0}^{\infty }\phi_{k}e^{-i\omega k}$ denote the discrete-time Fourier transform of the corresponding coarse-grained kernel. Then, in the limit as Δt→0, the following approximation holds:*

(A3)
$$\hat{\phi }\left(\nu \Delta t\right)=\tilde{\phi }\left(\nu \right)+O\left(\Delta t^{2}\right).$$

**Proof.** From (6) and (7), we obtain

(A4) $$\begin{array}{cc} \hat{\phi }\left(\nu \Delta t\right)\; & =\sum_{k=0}^{\infty }\phi_{k}e^{-i\nu k\Delta t}\\ \; & =\xi_{0}+\left(\xi_{1}-\xi_{0}\right)e^{-i\nu \Delta t}+\left(\xi_{2}-\xi_{1}\right)e^{-2i\nu \Delta t}+\cdots \\ \; & =\lim_{n\to \infty }\left[\sum_{k=0}^{n-1}\xi_{k}e^{-i\nu k\Delta t}\left(1-e^{-i\nu \Delta t}\right)+\xi_{n}e^{-i\nu n\Delta t}\right]\\ \; & =\lim_{n\to \infty }\left[\sum_{k=0}^{n-1}\int_{k\Delta t}^{(k+1)\Delta t}\varphi \left(t\right)e^{-i\nu k\Delta t}dt\cdot \frac{1-e^{-i\nu \Delta t}}{\Delta t}+\xi_{n}e^{-i\nu n\Delta t}\right], \end{array}$$

where $\varphi \left(t\right)=\int_{0}^{t}\phi \left(u\right)du$. To evaluate the integral, consider

(A5) $$\begin{array}{cc} \int_{k\Delta t}^{(k+1)\Delta t}\varphi \left(t\right)e^{-i\nu t}dt\; & =\int_{k\Delta t}^{(k+1)\Delta t}\varphi \left(t\right)\left[e^{-i\nu k\Delta t}-i\nu e^{-i\nu k\Delta t}\left(t-k\Delta t\right)+O\left(\Delta t^{2}\right)\right]dt\\ \; & =\int_{k\Delta t}^{(k+1)\Delta t}\varphi \left(t\right)e^{-i\nu k\Delta t}dt-Q_{k}+O\left(\Delta t^{3}\right), \end{array}$$

where

$$\begin{array}{c} Q_{k}=i\nu e^{-i\nu k\Delta t}\int_{0}^{\Delta t}\varphi \left(t+k\Delta t\right)tdt. \end{array}$$

By Taylor’s theorem, for each t∈[0,Δt], there exists c∈(kΔt,(k+1)Δt) such that

$$\varphi \left(t+k\Delta t\right)=\varphi \left(k\Delta t\right)+\dot{\varphi }\left(c\right)t=\varphi \left(k\Delta t\right)+\phi \left(c\right)t.$$

Note that *c* generally depends on *t*. Then, $Q_{k}$ is approximated as

$$\begin{array}{c} Q_{k}=i\nu e^{-i\nu k\Delta t}\int_{0}^{\Delta t}\left[\varphi (k\Delta t)+\phi (c)t\right]tdt=i\nu e^{-i\nu k\Delta t}\cdot \frac{\varphi (k\Delta t)}{2}\Delta t^{2}+O\left(\Delta t^{3}\right). \end{array}$$

Substituting into (A5), we obtain

$$\begin{array}{c} \int_{k\Delta t}^{(k+1)\Delta t}\varphi \left(t\right)e^{-i\nu k\Delta t}dt=\int_{k\Delta t}^{(k+1)\Delta t}\varphi \left(t\right)e^{-i\nu t}dt+i\nu e^{-i\nu k\Delta t}\cdot \frac{\varphi (k\Delta t)}{2}\Delta t^{2}+O\left(\Delta^{3}\right). \end{array}$$

Therefore, for n≫1 and $\Delta t∼n^{-1}$, it follows that

$$\begin{array}{cc} \sum_{k=0}^{n-1}\int_{k\Delta t}^{(k+1)\Delta t}\varphi \left(t\right)e^{-i\nu k\Delta t}dt\; & =\int_{0}^{n\Delta t}\varphi \left(t\right)e^{-i\nu t}dt+\frac{i\nu \Delta t}{2}\sum_{k=0}^{n-1}\varphi \left(k\Delta t\right)e^{-i\nu k\Delta t}\Delta t+O\left(\Delta t^{2}\right)\\ \; & =\int_{0}^{n\Delta t}\varphi \left(t\right)e^{-i\nu t}dt+\frac{i\nu \Delta t}{2}\int_{0}^{n\Delta t}\varphi \left(t\right)e^{-i\nu t}dt+O\left(\Delta t^{2}\right). \end{array}$$

Substituting back into (A4) yields

$$\begin{array}{cc} \hat{\phi }\left(\nu \Delta t\right)\; & =\lim_{n\to \infty }[\left(\int_{0}^{n\Delta t}\varphi \left(t\right)e^{-i\nu t}dt+\frac{i\nu \Delta t}{2}\int_{0}^{n\Delta t}\varphi \left(t\right)e^{-i\nu t}dt+O\left(\Delta t^{2}\right)\right)\\ \; & \times \left(i\nu +\frac{\nu^{2}\Delta t}{2}+O\left(\Delta t^{2}\right)\right)+\xi_{n}e^{-i\nu n\Delta t}]\\ \; & =\lim_{n\to \infty }\left(i\nu \int_{0}^{n\Delta t}\varphi \left(t\right)e^{-i\nu t}dt+\xi_{n}e^{-i\nu n\Delta t}\right)+O\left(\Delta t^{2}\right). \end{array}$$

The first term on the right-hand side can be further simplified as

$$\begin{array}{cc} i\nu \int_{0}^{n\Delta t}\varphi \left(t\right)e^{-i\nu t}dt\; & =-{\left[\varphi \left(t\right)e^{-i\nu t}\right]}_{0}^{n\Delta t}+\int_{0}^{n\Delta t}\dot{\varphi }\left(t\right)e^{-i\nu t}dt\\ \; & =-\varphi \left(n\Delta t\right)e^{-i\nu n\Delta t}+\int_{0}^{n\Delta t}\phi \left(t\right)e^{-i\nu t}dt. \end{array}$$

Consequently,

$$\begin{array}{cc} \hat{\phi }\left(\nu \Delta t\right)\; & =\lim_{n\to \infty }\left(\int_{0}^{n\Delta t}\phi \left(t\right)e^{-i\nu t}dt-\varphi \left(n\Delta t\right)e^{-i\nu n\Delta t}+\xi_{n}e^{-i\nu n\Delta t}\right)+O\left(\Delta t^{2}\right)\\ \; & =\int_{0}^{\infty }\phi \left(t\right)e^{-i\nu t}dt+O\left(\Delta t^{2}\right), \end{array}$$

where we have used the identity $\lim_{n\to \infty }\xi_{n}=\varphi \left(\infty \right)$. □

Now, we complete a proof of Theorem 2. According to Equation (17), the spectral density of the univariate coarse-grained Hawkes process is expressed as

$$f_{\Delta t}^{\left(cg\right)}\left(\omega \right)=\frac{\lambda \Delta t}{2\pi }\frac{1}{|1-\hat{\phi }{\left(\omega \right)|}^{2}}.$$

Substituting Equation (A3) into the expression above yields

$$\begin{array}{cc} f_{\Delta t}^{\left(cg\right)}\left(\nu \Delta t\right)\; & =\frac{\lambda \Delta t}{2\pi }\frac{1}{|1-\tilde{\phi }\left(\nu \right)+O\left(\Delta t^{2}\right){|}^{2}}\\ \; & =\frac{\lambda \Delta t}{2\pi }\left(\frac{1}{|1-\tilde{\phi }{\left(\nu \right)|}^{2}}+O\left(\Delta t^{2}\right)\right)\\ \; & =f^{\left(hw\right)}\left(\nu \right)\Delta t+O\left(\Delta t^{3}\right), \end{array}$$

thereby completing the proof of Theorem 2 in the univariate setting.

For comparison, consider the binned Poisson approximation introduced in Equations (1) and (2), wherein the discretized excitation kernel is defined by $\phi_{k}=\phi \left(k\Delta t\right)\Delta t$ for k=1,2,…. The discrete-time Fourier transform of this kernel is given by

$$\begin{array}{c} \hat{\phi }\left(\nu \Delta t\right)=\sum_{k=1}^{\infty }\phi \left(k\Delta t\right)e^{-i\nu k\Delta t}\Delta t=\tilde{\phi }\left(\nu \right)+O\left(\Delta t\right), \end{array}$$

as Δ→0. Observe that the approximation order is lower than that of Equation (A3). Consequently, the spectral density of the binned Poisson approximation is asymptotically given by

$$f_{\Delta t}^{\left(po\right)}\left(\nu \Delta t\right)=f^{\left(hw\right)}\left(\nu \right)\Delta t+O\left(\Delta t^{2}\right).$$

## Appendix B. Second-Order Properties of the Stationary Hawkes Process

This appendix provides a concise summary of the second-order statistical characteristics of the stationary Hawkes process. We present only those results pertinent to the analysis in this paper, and refer the reader to [22,28,29] for detailed derivations.

- Spectral Density Matrix of the Stationary Hawkes Process:
  (A6) $$F^{\left(hw\right)}\left(\nu \right)=\frac{1}{2\pi }{\left(I-\tilde{\Phi }\left(\nu \right)\right)}^{-1}\mathrm{diag}\left(\lambda \right){\left(I-{\tilde{\Phi }}^{T}\left(\nu \right)\right)}^{-1},\;\nu \in \left(-\infty ,\infty \right),$$
  where $\tilde{\Phi }\left(\nu \right)=\int_{0}^{\infty }\Phi \left(t\right)e^{-i\nu t}dt$ denotes the Fourier transform of the excitation kernel matrix, and
  $$\lambda =E\left[\lambda \left(t\right)\right]={\left(I-A\right)}^{-1}\mu ,$$
  represents the stationary (mean) intensity.
- Expected Value of the Binned Stationary Hawkes Process:
  (A7) $$E\left[X_{k}\right]=\lambda \Delta t={\left(I-A\right)}^{-1}\mu \Delta t.$$
- Spectral Density Matrix of the Binned Stationary Hawkes Process:
  (A8) $$\begin{array}{c} F_{\Delta t}^{\left(hw\right)}\left(\omega \right)=\Delta t\sum_{k=-\infty }^{\infty }{\mathrm{sinc}}^{2}\left(\frac{\omega +2\pi k}{2}\right)F^{\left(hw\right)}\left(\frac{\omega +2\pi k}{\Delta t}\right),\;\omega \in \left[-\pi ,\pi \right). \end{array}$$

To evaluate (A8) in the limit as Δt→0, we employ the following asymptotic expansion:

$$\mathrm{sinc}\left(\frac{\nu \Delta t+2\pi k}{2}\right)=\left\{\begin{array}{cc} 1-\frac{\nu^{2}\Delta t^{2}}{12}+O\left(\Delta t^{4}\right), & k=0,\\ \frac{\nu^{2}\Delta t^{2}}{4\pi^{2}k^{2}}+O\left(\Delta t^{3}\right), & k\neq 0. \end{array}\right.$$

Substituting into (A8) yields the approximation:

$$\begin{array}{cc} F_{\Delta t}^{\left(hw\right)}\left(\nu \Delta t\right)\; & =\left(1-\frac{\nu^{2}\Delta t^{2}}{12}+O\left(\Delta t^{4}\right)\right)F^{\left(hw\right)}\left(\nu \right)\Delta t\\ \; & +\sum_{k\neq 0}\left(\frac{\nu^{2}\Delta t^{2}}{4\pi^{2}k^{2}}+O\left(\Delta t^{3}\right)\right)F^{\left(hw\right)}\left(\nu +\frac{2\pi k}{\Delta t}\right)\Delta t\\ \; & =F^{\left(hw\right)}\left(\nu \right)\Delta t+O\left(\Delta t^{3}\right). \end{array}$$

## Appendix C. Power-Law Distribution

A power-law distribution for waiting times is defined as

$$g\left(t\right)=\frac{\beta \gamma }{{\left(1+\beta t\right)}^{1+\gamma }},\;t\geq 0,$$

for β>0 and γ>0. It is well-known that the moments of a power-law distribution exist and are finite for all orders strictly less than the exponent γ. In particular, the expected waiting time is finite if γ>1, and is given by

$$\int_{0}^{\infty }tg\left(t\right)dt=\frac{1}{\beta (\gamma -1)}.$$

The corresponding coarse-grained kernel is given by

$$g_{k}=\left\{\begin{array}{cc} 1-\left[{\left(1+\beta \Delta \right)}^{1-\gamma }-1\right]/\beta \left(1-\gamma \right)\Delta , & k=0,\\ -\left[{\left\{1+\beta \left(k+1\right)\Delta \right\}}^{1-\gamma }-2{\left\{1+\beta k\Delta \right\}}^{1-\gamma }+{\left\{1+\beta \left(k-1\right)\Delta \right\}}^{1-\gamma }\right]/\beta \left(1-\gamma \right)\Delta , & k=1,2,\ldots , \end{array}\right.$$

for γ≠1, and

$$g_{k}=\left\{\begin{array}{cc} 1-\log(1+\beta \Delta )/\beta \Delta , & k=0,\\ -\left[\log\{1+\beta (k+1)\Delta \}-2\log\{1+\beta k\Delta \}+\log\{1+\beta (k-1)\Delta \}\right]/\beta \Delta , & k=1,2,\ldots , \end{array}\right.$$

for γ=1.

Figure A1 illustrates the power spectral density (PSD), cross-spectral density (CSD), auto-covariance, and cross-covariance of the binned Hawkes process (blue dotted lines), the coarse-grained Hawkes process (red lines), and the binned Poisson approximation (green lines), respectively. Figure A2 presents the information loss associated with the coarse-grained Hawkes process (red lines) and the binned Poisson approximation (green lines), respectively. These results are qualitatively consistent with those obtained using the exponential kernel, as shown in Figure 1 and Figure 2.

Figure A3, Figure A4, Figure A5 and Figure A6 summarize the results of parameter estimation in scenarios where the excitation kernels follow power-law distributions. The MC-EM algorithm was omitted, as the power-law kernel is not supported by the publicly available implementation [25]. As with the exponential kernel case shown in Figure 3, Figure 4, Figure 5 and Figure 6, the proposed method consistently outperforms both the binned Poisson MLE and the INAR(*p*) method, particularly with respect to reducing estimation bias.
